# Community-Based Health Planning and Services Plus programme in Ghana: A qualitative study with stakeholders in two Systems Learning Districts on improving the implementation of primary health care

**DOI:** 10.1371/journal.pone.0226808

**Published:** 2020-01-08

**Authors:** Margaret Kweku, Hubert Amu, Adam Awolu, Martin Adjuik, Martin Amogre Ayanore, Emmanuel Manu, Elvis Enowbeyang Tarkang, Joyce Komesuor, Geoffrey Adebayo Asalu, Fortress Yayra Aku, Nuworza Kugbey, Fidelis Anumu, Laud Ampomah Boateng, Justine Sefakor Alornyo, Roland Glover, Timothy Letsa, Ayaga A. Bawah, Nicholas S. Kanlisi, John Koku Awoonor-Williams, James F. Phillips, John Owusu Gyapong

**Affiliations:** 1 School of Public Health, University of Health and Allied Sciences, Hohoe, Ghana; 2 Institute of Health Research, University of Health and Allied Sciences, Ho, Ghana; 3 Volta Regional Health Directorate, Ghana Health Service, Ho, Ghana; 4 Regional Institute of Population Studies, University of Ghana, Legon, Accra, Ghana; 5 Mailman School of Public Health, Columbia University, New York, New York, United States of America; 6 Policy Planning Monitoring and Evaluation Division, Ghana Health Service, Accra, Ghana; Tabriz University of Medical Sciences, IR Iran, ISLAMIC REPUBLIC OF IRAN

## Abstract

**Background:**

In 1999, Ghana introduced the Community-Based Health Planning and Services (CHPS) as the key primary health care strategy. In this study, we explored the challenges, capacity development priorities, and stakeholder perspectives on improving the CHPS concept as it has been fraught with a myriad of challenges since its inception. Our study is the outcome of the national programme for strengthening the implementation of CHPS Initiative in Ghana (CHPS+) introduced in 2017.

**Methods:**

This exploratory research was a qualitative study conducted in two Systems Learning Districts (SLDs) of CHPS+ in the Volta Region of Ghana from March to May, 2018. Four focus group discussions and two general discussions were conducted among 60 CHPS+ stakeholders made up of health workers and community members. Data analyses were conducted using conceptual content analysis. Statements of the participants were presented as quotes to substantiate the views expressed.

**Results:**

Negative attitude, high attrition, inadequacy and unavailability of health professionals at post when needed were challenges associated with the health professionals. Late referrals, lack of proper community entry and engagement, non-availability of essential logistics, distance of CHPS compounds from communities, and inadequate funding were challenges associated with the health system. Lack of community ownership of the CHPS programme, lack of security at CHPS compounds, and late reporting of cases by the community members were also realised as challenges emanating from the community members. Priority areas for capacity development of health workers identified included logistics management, community entry and engagement, emergency delivery, managing referrals at the CHPS level, and resuscitation of newborns.

**Conclusion:**

Health-worker, community, and health systems-based challenges inhibit the implementation of CHPS in Ghana. Capacity development of health professionals and continuous community engagement are avenues that can improve implementation of the programme.

## Introduction

Primary Health Care (PHC) is an essential health care which is grounded on socially acceptable and scientifically sound methods that are universally accessible to individuals and families with their full participation at costs that countries and communities can afford in a spirit of self-determination and self-reliance [1,2]. It encompasses health services organised based on the needs and expectations of the people, leadership that enhances collaborative policy dialogue, public policies that incorporate health into all sectors, increased stakeholder involvement and universal health coverage to reduce exclusion and social disparities in health [3–6].

In Ghana, the key PHC programme is the Community-based Health Planning and Services (CHPS) [7]. Introduced in 1999 [8], CHPS is a national strategy geared towards the delivery of crucial community-based health services involving service delivery and health planning with communities [9]. The primary focus of CHPS is to bring health services close to communities. CHPS’ aim is to move health services to community locations, develop sustainable volunteerism and community health action, empower women and vulnerable groups, and improve health provider, household, and community interaction [9].

Ghana has made several efforts at achieving universal health coverage using CHPS and other interventions including health insurance [7,9]. The country for instance, abolished user fees at the PHC level in the public sector through the introduction of a National Health Insurance Scheme (NHIS) (Act 650 of Parliament) [10,11], which began full implementation in 2005 [12,13]. The implementation of the NHIS at public health facilities and CHPS at the community level helped to promote equity and increased access to basic health services for the poor [14,15].

As a result of the increase in the number of people accessing basic health services due to the introduction of the CHPS concept, there were reduced infant and child mortalities through immunisation, reduced water borne diseases, increased breastfeeding, and increased household involvement in treatment of diarrhoea, though not to desirable levels [4]. The CHPS programme did not achieve desirable results due to the general misunderstanding of the CHPS strategy which has made its implementation to largely focus on building of infrastructure and the provision of clinical services [16]. There was, therefore, the need to implement new strategies aimed at revamping the CHPS concept to achieve its core objective of bringing health care to the doorstep of communities. This need led to the implementation of a new National Programme for strengthening the implementation of CHPS Initiative in Ghana (CHPS+) in 2017.

Ghana is a West African country with a total land area of 238,533 km^2^ [17]. The country’s population in 2019 based on projections from the 2010 population and housing census of Ghana is 30,280,811. This is made up of 14,888,808 males and 15,392,003 females [18]. There are 3217 functional health facilities in the country. Out of this number, four are teaching hospitals. Also, there are nine regional hospitals, three psychiatric hospitals, 11 polyclinics, 96 public hospitals, 59 Christian Health Association of Ghana (CHAG) hospitals, 156 private hospitals, 22 quasi-government hospitals, and 10 Islamic hospitals.

## The CHPS+ project

CHPS+ is a collaboration project carried out by the Ghana Health Service (GHS), the Mailman School of Public Health of Columbia University, USA, the University of Ghana’s Regional Institute of Population Studies, Ghana, the University of Health and Allied Sciences (UHAS), School of Public Health, and the University for Development Studies (UDS) with funding from the Doris Duke Charitable Foundation, USA [7,17]. CHPS+ is a scale up of the Ghana Essential Health Interventions Programme (GEHIP) which was piloted in three administrative districts of the Upper East Region in Ghana [7]. After a successful piloting, it was then scaled up to cover more regions in Ghana and thus leading to CHPS+ [7].

The mandate of UHAS (the institution which led the conduct of the current research) under the CHPS+ project is to develop capacity of frontline health staff in the Volta Region through the provision of short and long-term training of the staff. Before this could be achieved, however, there was the need for an exploratory research to assess the training needs of the frontline health workers, which partly resulted in the conduct of the present study. The focus of CHPS+ in the Volta Region is primarily targeted at two administrative districts (Nkwanta South and Central Tongu) selected by the Volta Regional Health Directorate (VRHD) and labeled ‘Systems Learning Districts’ (SLDs). This, therefore, also influences the choice of the two districts for the present study.

Established in 2011 by Act 828 of the Parliament of Ghana (became operational in 2012) [19], UHAS was selected for the project because, it is a new health university focused on problem based learning which is an approach essential to the implementation of CHPS+. The Volta Region was also chosen under the project because it had one of the highest under-five mortality (61/1000) and fertility (4.8) rates in Ghana [20].

There are three implementation phases required of UHAS under the CHPS+ project. This study is the outcome of the second phase which entails planning and conducting research among the various stakeholders (frontline health workers and community members) in the SLDs in order to ascertain the capacity development/training needs of the frontline health workers’ (CHOs, staff at the sub-district level, and those at the district level). The primary objective of this study was, thus, to ascertain frontline health workers’ capacity development/training needs in order to develop capacity development courses based on those needs. We also sought to understand the challenges which confronted health workers in their implementation of CHPS and sought from them, their perspectives on improving the implementation of CHPS.

## Conceptual framework

**Fig 1:**
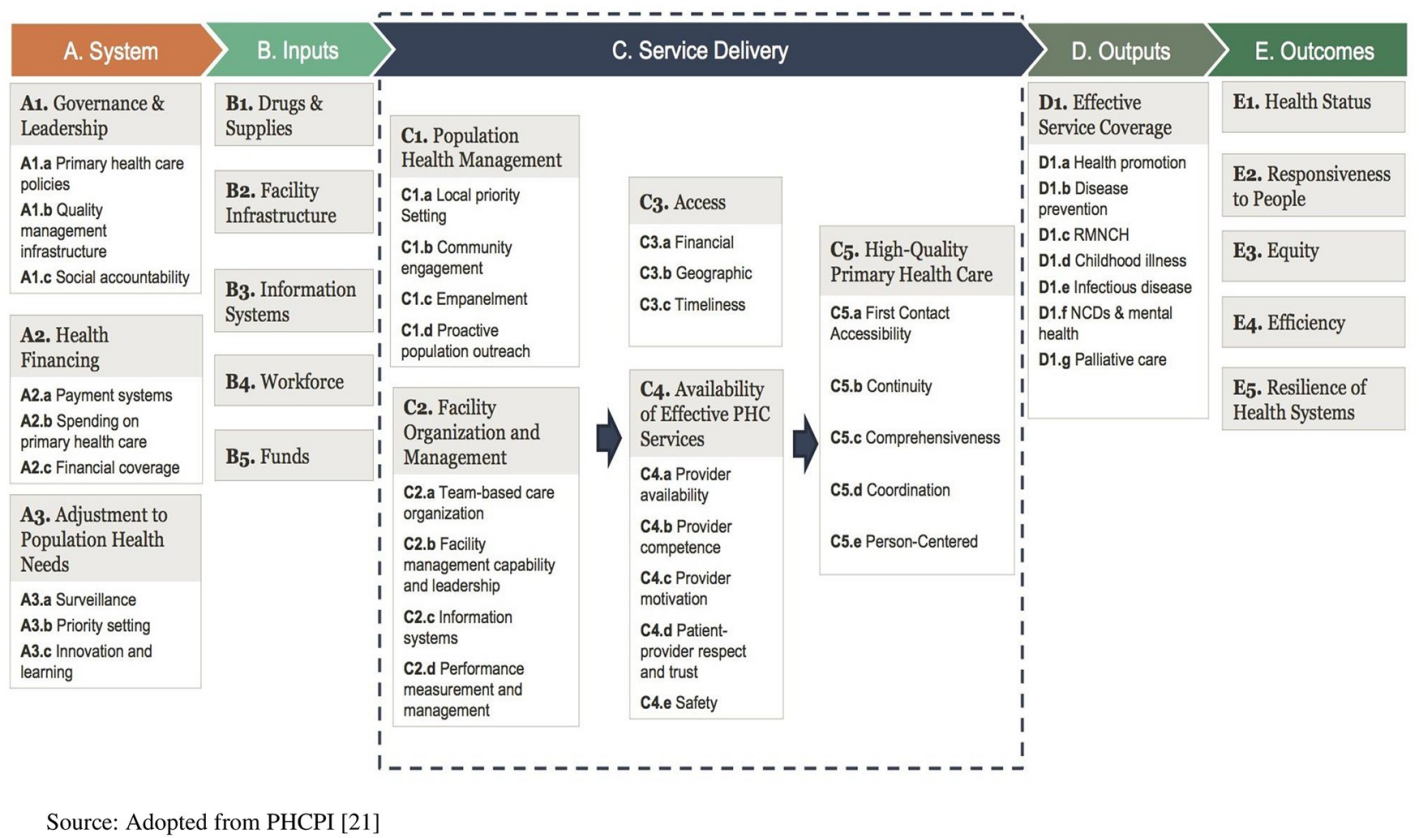
Conceptual framework.

The study adopted the conceptual framework by the Primary Health Care Performance Initiative (PHCPI) [21]. Tenets of the framework comprise the system (Is PHC prioritised in the country’s health system?), key inputs (Does the PHC system offer sufficient facilities, health care professionals, and supplies?), service delivery (Are services accessible and effectively organised, managed, and coordinated to deliver high quality care?), outputs (Does the PHC system provide the essential services you need throughout each phase of life?) and outcomes (Does the PHC system efficiently deliver better outcomes and greater equity?) [22,23]. From Fig 1, the framework flows from left to right, which is synonymous to other input-process-output-outcome logic models. A focus on service delivery, however, differentiates the framework from other input-process-output-outcome frameworks.

The framework was adopted to underpin the present study because of its components which are intrinsic in our study. These include availability and access to effective CHPS services, people-centered care, community engagement, health promotion, health financing, financial and geographic access to health care as well as timeliness of care provided, disease prevention, equity, efficiency, organization, and management.

## Materials and methods

### Setting

**Fig 2:**
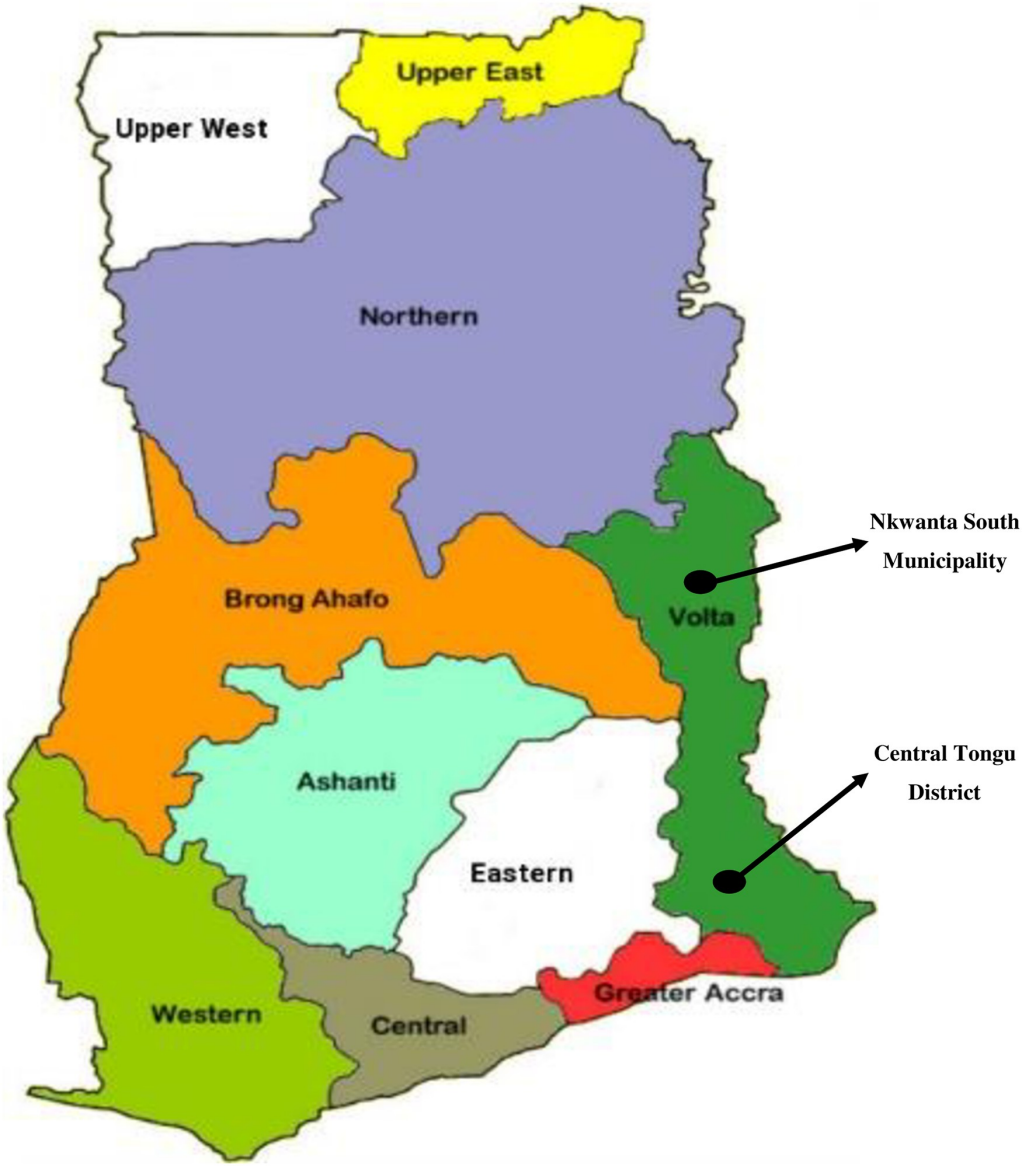
Map of Ghana showing the study areas.

The present study was conducted in the two CHPS+ SLDs in the Volta Region (Nkwanta South Municipality and Central Tongu District) of Ghana from March to May 2018. The region has a total of 326 health institutions out of which 242 are administered by the GHS, 18 are mission owned, one is quasi-government and 65 are privately owned [17]. As of 2017, the population of the region according to the Ghana Statistical Service based on the 2010 census figures was 2,491,293 [18]. Out of this, males constituted 1,223,722 while females formed 1,239,279. According to the Ghana Statistical Service [18], the population of Central Tongu and Nkwanta South were 112,095 and 57,290 respectively in 2017. The population of Central Tongu comprises 54,990 males and 57,105 females while the population of Nkwanta South is made up of 28,102 males and 29,188 females. Fig 2 presents a map of the study setting.

The choice of the two districts for the CHPS+ project (which the current study adopted) was because that they belong to two different ecological zones; central Tongu in the coastal zone and Nkwanta South in the Savannah zone. It was also because the region is usually classified into a Northern-Southern dichotomy. From this dichotomy, Nkwanta South was selected from the Northern zone while Central Tongu was chosen from the Southern zone.

### Design

The study was a qualitative research and an exploratory approach was adopted in conducting the study [24]. A total of 60 stakeholders made up of frontline health workers and community stakeholders were included in four focus group discussions (FGDs) [24]. Service providers recruited for the study included midwives, nurses, medical doctors, directors of health services, public health officers, and community health officers. Community members were also made up of chiefs, assembly members, community health volunteers, and ordinary community members. The various groups of health workers and community members were in each of the respective interviews for health workers and community members.

Ethical clearance for the CHPS+ Project under which the study was conducted, was provided by the Ghana Health Service Ethics Review Committee (Number: GHS-ERC:04/01/2017). Written informed consent was obtained from the participants before including them in the interviews. This was achieved by giving them informed consent forms to sign/thumbprint.

### Data collection instruments and procedures

Scorecards, FGD guides, and socio-demographic screening sheets were used as the main instruments for data collection. Three scorecards were deployed; Cards 1, 2, and 3. Card 1 was a scoring matrix for priority capacity development needs of service providers and had three sections; issue, priority, and reasons. Thus, in this card, participants were required to indicate the issues which constituted capacity development needs for them. The needs indicated were then prioritised using numerical scores of 1,2,3,4,5, etc. After this, participants were required to give reasons for the levels of priority given to the various issues which constituted their capacity development needs (See Table 1).

**Table 1:** Scoring matrix for priority capacity development needs of service provider.

No	Issue	Priority	Reasons
			
			
			
			
			
			
			
			
			
			

Card 2 was a scoring matrix for standard indicators of health care delivery adopted from the International Organisation on Migration (IOM). This was deployed among the health workers. Indicators included in this card were safety, effectiveness, efficiency, time, equitability, and patient-centeredness. For each of these indicators, the participants were asked to score them based on a likert scale of ‘very bad’, ‘bad’, ‘Just OK’, ‘Good’, and ‘Very good’ (See Table 2).

**Table 2:** Scoring matrix for standard indicators of health care delivery.

Indicator	Very bad	Bad	Just ok	Good	Very good
**Safety**					
**Effectiveness**					
**Efficiency**					
**Time**					
**Equitable**					
**Patient-centered**					

Card 3 was a scoring matrix for indicators developed for community members. The indicators comprised waiting time, health workers’ communication with patients, health workers’ availability, services rendered, distance of facility to community, and drugs availability. Just like Card 2, the participants were asked to then score the indicators based on a likert scale of ‘very bad’, ‘bad’, ‘Just OK’, ‘Good’, and ‘Very good’ (See Table 3).

**Table 3:** Scoring matrix for indicators developed for community members.

Indicator	Very Bad	Bad	Just OK	Good	Very Good
Waiting time					
Health workers’ communication					
Health workers availability					
Services rendered					
Distance of facility to community					
Drugs availability					

The cards were given to the participants to fill in/tick. Those who were not literates were supported by the research team to respond to the cards. Thus, they told the team members what their options/issues were, and the team members in turn wrote them in the respective slots for them. After the score cards were filled, separate focus group discussions (FGDs) were conducted for community members and health professionals respectively, in the two districts. The FGDs were largely based on the issues contained in the score cards. Thus, each FGD afforded the individuals in the group the opportunity to make known to other group members, the issues that were of priority to them, how they rated and prioritised them, and why they chose the things they considered priority areas for them. As such, the results of the scorecards were not directly analysed as they only aided the conduct of the FGDs. Overall, 4 FGDs were conducted; two at Nkwanta South and the other two at Central Tongu. They comprised one FGD each for health workers and community members in each of the districts. The FGD for health workers at Nkwanta South was labelled as ‘FGD 1’ while that for community members was labelled as ‘FGD 2’. For Central Tongu, the FGD for health workers was labelled as ‘FGD 3’ and that for community members labelled ‘FGD 4’.

The focus groups for community members had 14 and 13 participants respectively in the Nkwanta South Municipality and Central Tongu District. Key community stakeholders involved were chiefs, assembly members, linguists, queen mothers, and community health volunteers. The FGDs for health workers had 17 and 16 participants respectively in the Nkwanta South Municipality and Central Tongu District. Key stakeholders included in the health workers’ FGDs included District Directors of Health, District Public Health Nurses, Community Health Nurses/Officers, District Disease Control Officers, and CHPS Coordinators.

**Fig 3:**
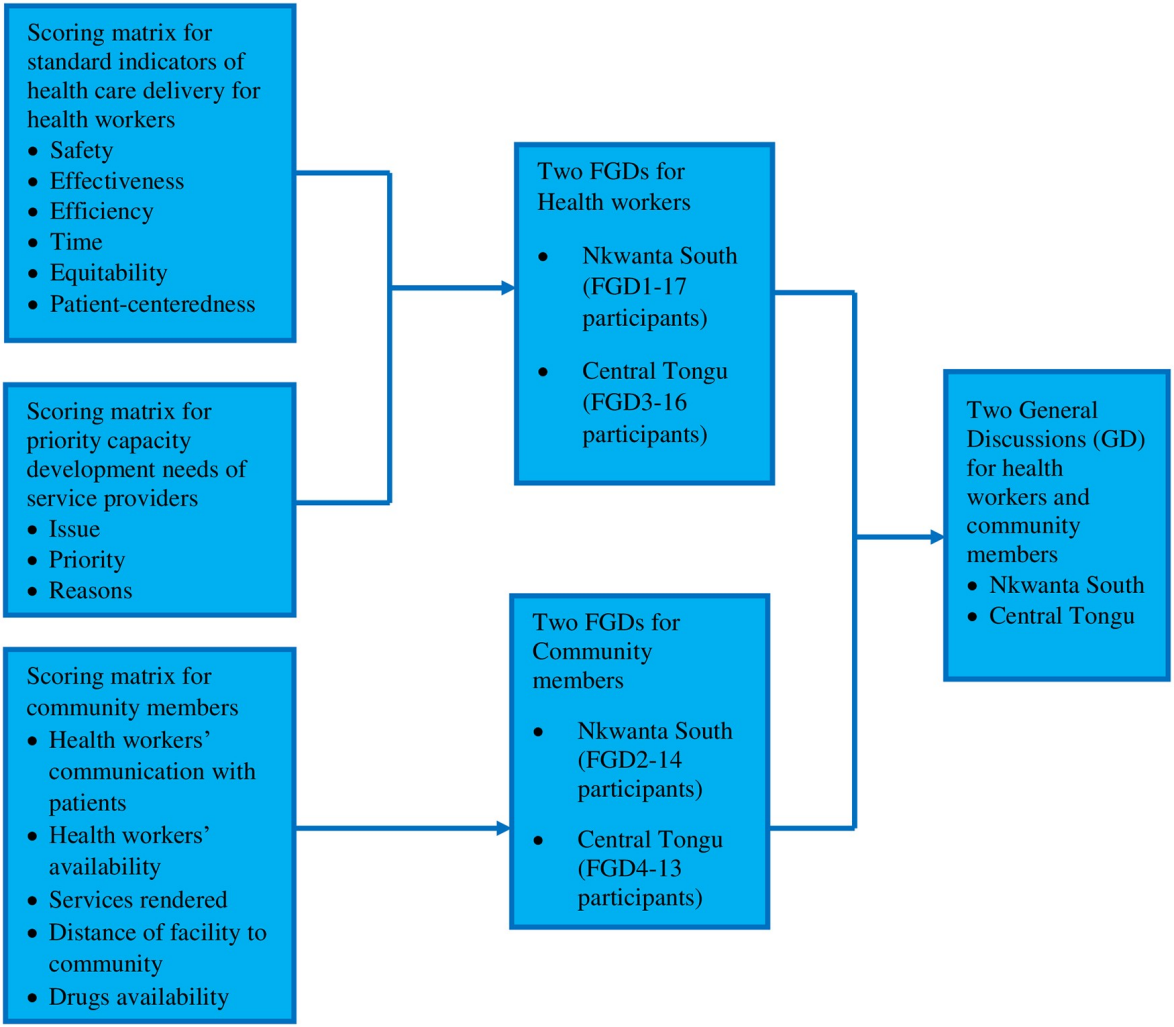
Flow chart of scorecards, FGDs and GDs of the study.

After the separate FGDs, an interface meeting was held in each of the districts where community members and the health workers were all brought together. In this meeting, community members expressed their concerns and ratings for health services delivered at the various CHPS compounds. A critical component of the interface meeting was that the GHS team and community representatives expressed their concerns about each other and both teams had the opportunity to respond to concerns raised. The interface meetings in Nkwanta and Central Tongu are labelled as GD (General Discussion) 1 and GD2 respectively. Fig 3 is a flowchart of the score cards, FGDs, and GDs conducted.

The socio-demographic information screening sheet used contained the Systems Learning District, sex, age, religion, ethnicity, marital status, and role in CHPS implementation. The Systems Learning District variable was divided into two; Central Tongu and Nkwanta South. The sex of the participants was also divided into two attributes; Male and Females. Age was left open in the instrument. It was, however, recategorised after data collection into five sub-sections; 20–29, 30–39, 40–49, 50–59, and 60+. Religion was also left open, but categorised after the data collection into Ewe, Akan, and ‘other’. Marital status was also left open but grouped into; Never married, Married; and Widowed, after the data collection. The role of the participant in CHPS implementation was also divided into two; health worker; and community member/stakeholder.

Data collection in the Nkwanta South Municipality was conducted on Friday, March 9, 2018, at Nkwanta, while that of Central Tongu District was carried out on Thursday, March 15, 2018, at Adidome. At each of the systems learning districts, the interviews were conducted by six of the seven researchers who were assisted by the six research assistants. The interviews lasted between one hour 30 minutes and two hours.

The research assistants supported the researchers by holding audio recorders during the interviews in addition to taking notes of the process. They also together with the researchers, supported the participants who were not literate, in filing the score cards. The researchers and research assistants prior to the data collection, were all taken through a two-day training to orient them on the purpose of the study, questions in the data collection instruments, the roles each of them was expected to play in the data collection process. The researchers were selected due to their experience in conducting qualitative interviews. The research assistants were also all full-time research assistants employed by the University of Health and Allied Sciences. Their recruitment for the data collection was because they had been involved in several previous projects carried out by the school of public health.

### Analysis

Manual thematic analysis was done by first of all, assigning preliminary codes to the data in order to describe the content. Patterns or themes in the codes which were mainly emergent across the different interviews were then developed. The themes realised were named and reviewed. To ensure inter-rater reliability and reduce biases in coding and development of the themes, the codes and themes were initially developed independently by two of the researchers who were involved in the data collection. After this, they compared their notes and agreed on common codes and themes to be used in the final analysis. In cases where there were disagreements, a third researcher who is a qualitative research professor in the school of public health, was brought in to help decide the appropriate code/theme to be used. Statements of the participants are presented as quotes to substantiate the views expressed. Frequencies and percentages are, however, used to present the socio-demographic characteristics (age, sex, marital status, religion, and role in CHPS implementation) of the participants.

## Results

The results are organised based on the socio-demographic characteristics of the participants and the themes that emerged from the study.

### Socio-demographic characteristics of participants

Table 4 presents the socio-demographic characteristics of participants. A total of 31 participants constituting 48.3% were from the Nkwanta South, whiles 29 representing 51.7% were from Central Tongu. Males from both districts constituted 55%. Participants were generally in their 30s (33.3%) and 40s (35%), with Christians constituting (85%), Ewes making up 73.3% and 80% being married. Community stakeholders involved in the study constituted 45%, whiles health workers formed 55%.

**Table 4:** Socio-demographic characteristics of study participants.

Variable	Frequency	Percentage (%)
**Systems Learning District**		
Nkwanta	31	51.7
Central Tongu	29	48.3
**Sex**		
Male	33	55.0
Female	27	45.0
**Age (in completed years)**		
20–29	<5	5.0
30–39	20	33.3
40–49	21	35.0
50–59	9	15.0
60+	7	11.7
**Religion**		
Christian	51	85.0
Muslim	<5	3.3
African Traditionalist	7	11.7
**Ethnicity**		
Ewe	44	73.3
Akan	13	21.7
Other	<5	5.0
**Marital status**		
Never married	8	13.3
Married	48	80.0
Widowed	<5	6.7
**Role in CHPS implementation**		
Health worker	33	55.0
Community member/stakeholder	27	45.0
**Total**	**60**	**100.0**

### Thematic results

Three major themes emerged from the FGDs and the GDs. The first were challenges associated with the implementation of CHPS, the second theme was about priority areas of capacity development for frontline health workers in the implementation of CHPS, and the third centered on stakeholder perspectives on improving the implementation of CHPS. Table 5 presents the thematic table of the study.

**Table 5:** Thematic table.

Main theme	Sub-theme code
Challenges	Health-worker based challenges negative attitude of health workers high attrition rates of CHOs/Community Health Nurses (CHNs) inadequacy and/or unavailability of CHOS/CHNs at post when needed
	Community-based challenges lack of community ownership of the CHPS programme lack of security at CHPS compounds late reporting of cases by the community members
	Health systems-based challenges late referrals lack of proper community entry and engagement non-availability of essential logistics for running CHPS distance of CHPS Compounds from communities inadequate funding/lack of resources
Priority areas for capacity development	Logistics management
	Community entry and engagement
	Emergency delivery
	Communicative skills
	Conflict resolution and management
	Data management
	Management and leadership skills
	Integrated management of neonatal and childhood illnesses Management of non-communicable and communicable diseases
	Managing referrals at the CHPS level
	Managing neglected tropical diseases in the communities
	Integrated diseases surveillance and response
	Logistics management
	Resuscitation of newborns
Stakeholder perspectives on improving CHPS implementation	Addressing the challenge of community lack of ownership of CHPS programmes
	Addressing the lack of community entry and engagement
	Addressing the non-availability of medicines
	Self-help
	Communities support for health workers in CHPS implementation

### Challenges associated with the implementation of CHPS

The main challenges identified were grouped into three main sub-themes; health-worker based, community-based, and health systems-based challenges.

#### Health worker-based challenges

Health worker-based challenges were defined as challenges which emanated from the frontline health workers rendering PHC to the communities through CHPS. The major health worker-based challenges, which emerged from the discussions comprised negative attitude of health workers, high attrition rates of CHOs/Community Health Nurses (CHNs), as well as the inadequacy and/or unavailability of CHOS/CHNs at post when needed.

Regarding negative attitude of health workers, participants in the two FGDs for community members indicated that sometimes when they visited the CHPS compounds for services, the nurses spoke to them. The following quote summarises their responses:

What I have seen about the nurses at our place is that [errm] for the community (health) nurses, it is left with something little for them (improvements required). It is like sometimes when you go there (CHPS compound), the nurses don’t treat you nicely. They only talk to you somehow (rudely)

(FGD 2, Male, 39 years).

High attrition rate was generally reported by both health workers and community members in their respective FGDs as a major challenge bedeviling the smooth implementation of CHPS in the two SLDs. According to the participants, CHOs posted to the CHPS compounds do not take time to get used to the community before they leave; either transferred elsewhere or go for further education. The community members for instance, bemoaned the situation where sometimes, the communities tend to really like the services provided by the CHOs and when level of utilisation of the PHC starts increasing due to that then the service provider leaves abruptly. A 47-year-old participant in FGD 2 for instance had this to say:

What I want to put before us is that you see that the nurses, when they come small like this, it won’t be long then they say they’ve taken them away. Maybe within the time she has stayed in the village, she and the people are getting to be free or something like that and they are liking the services she is giving them, then you hear, they will say [ooh] she should go

(FGD 2, Male, 47 years).

Regarding inadequacy of the CHOs, it was revealed that the average number of CHOs per CHPS compound is two. Community members particularly complained that the number is woefully inadequate and added that despite the inadequacy, they are also usually not available to attend to patients who need their services due to various reasons. Some of them for instance, are always not around during weekends for them to attend to those who need care. The following quotes summarise their responses.

The nurses are only three, but they are usually not around during weekends… Usually, people walk long distances and come there (CHPS compound) only to meet no one … it is not good at all. Because, when people come and they don’t meet the nurses, they will stop coming. There was a time we called one of the nurses to inform him that people were looking for him, but he got angry with us that we didn’t employ him

(FGD 4, Male, 46 years).

#### Community-based challenges

Community-based challenges comprise those bottlenecks which emanated from the communities and hindered the efficient implementation of CHPS. The major community-based challenges which were identified through the FGDs were lack of community ownership of the CHPS programme, lack of security at CHPS compounds, and late reporting of cases by the community members.

Regarding lack of community ownership of CHPS programmes, the health workers indicated that most of the time, when they visit the communities to carry out CHPS activities, there is usually some level of apathy among the community members towards their services. According to them, this was largely because the communities did not see the CHPS as their own and therefore did not want to participate in activities relating to it. It was revealed from the FGDs that a major challenge leading to the lack of ownership of CHPS programme by the communities is that they are not properly informed on the activities before the health workers go around to carry them out. The following quotes summarise the discussions in this regard:

The communities do not own the programme that is being rolled up (CHPS). So, they don’t understand and know that the programme is for them. So, because of that they don’t give the maximum support to our health workers on the field (CHOs)

(FGD 1, Female, 34 years).

Concerning lack of security at CHPS compounds, it emerged from the FGDs that the provision of security for the CHOs and the CHPS compounds was the responsibility of the various communities in which the compounds are situated. The community members, however, reneged on this responsibility. The community members felt the health system is supposed to give the security personnel remunerations after they have successfully placed the individuals as security guards at the CHPS compounds, and because that was not happening, the security guards also decided to stop working. Some of the highlights of this challenge are presented in the quotes below:

When we talk of CHPS, provision of security at CHPS compounds is the sole responsibility of the chiefs and elders of the community. But for the security person, doing it as a voluntary work or something like that without any motivation is a problem…He comes to watch over the facility and the staff over there (CHPS compound). So, they should at least give him a token (money/allowance) at the end of the month or even help him on his farm. But this is missing

(FGD 2, Male, 53 years).

Regarding late reporting of cases, it was realised from both FGDs conducted among the health workers that community members usually waited until their health conditions aggravated before they turned up for care at the CHPS compounds. Most of these people were pregnant women who tried giving birth at home but realised it was not successful.

What I have realised personally is that most people report cases very late. These are usually people who try giving birth at home and they don’t succeed; not people who have made up their minds that “I will deliver in the health facility”

(FGD 1 Male, 36 years).

#### Health systems-based challenges

Health systems-based challenges were those challenges, which originated from the health system and inhibited the effective implementation of CHPS at the two SLDs. The major challenges, which emerged from the FGDs were: late referrals, lack of proper community entry and engagement, non-availability of essential logistics for running CHPS, distance of CHPS Compounds from communities, and inadequate funding/lack of resources.

Concerning late referrals, it was realised from the FGDs that referrals from the lower levels (CHPS compounds) were usually done late. Complications, therefore, occurred before patients reached the sub-district/district levels for further care. The following quote reiterates this finding:

I will talk about late referral. Sometimes, when cases coming from the sub-district levels, before they get to the hospital, there are sometimes complications and this is largely because there are usually some kind of delays in referring the patients. So, this is a major challenge for us here (at the district hospital)

(FGD 1, Female, 35years).

Regarding lack of proper community entry and engagement, it was realised that a major reason for the lack of ownership of the CHPS programme by the community members emanated mainly from health workers usually not carrying out appropriate community entry processes to engage the community leaders and their members on health programmes. When programmes are eventually rolled out, the people then decide not to participate in them. Due to lack of community entry and stakeholder engagement, Traditional Birth Attendants (TBAs) are also usually unwilling to refer cases to the health system as they do not feel recognised. The following quotes summarise these findings:

What I wrote was community entry. It is very important when we are talking about CHPS implementation. Yeah! It’s like we are doing it (implementing CHPS) but the community entry is not there. Yea! we just post stuff to the community without engaging those we are supposed to engage in the community, especially the chiefs and his elders and other factors in the community

(FGD 1, Male, 33 years).

In fact, everything boils down to community entry, and because there is no (good) relationship between the health staff and the TBAs, it is like the TBA is aware of a health staff in the community, but will not refer (them to the health worker). When the cases get bad where they cannot sustain it (unable to handle the delivery) then they shift it to the health staff. At times, the TBAs refuse to even do the referral.

(FGD 1, Female, 36 years).

With regards to non-availability of essential logistics for delivering CHPS, it was realised that medicines needed for the treatment of basic conditions before referral to higher levels of care, according to both community members and the health workers, were usually non-existent. For the community members, their major challenge was when they were asked to always buy medicines required for the ailments on their own, when they felt the health system could have done so for them, so that once they come to the facility, they would just be treated and given their medications. Vaccine fridges and motor bikes for making community rounds and outreaches according to the health workers, are usually non-existent. The following quotes summarise these results:

My priority area is the non-availability of basic and critical logistics. Because you have an instance where a CHPS compound or health facility will be operationalized, but there is no vaccine fridge or other critical logistics for the health facility to be able to perform up to expectation

(FGD 1, Male, 35 years).

When it gets to sometimes, when you go, when you fall sick and go, there is no medicine and they will write that you should go and buy. Our place too, we don’t have any drugstore in the town. Unless, you go to Breweniase or Nkwanta before you will get the medicine to buy. So, it is something that we really need

(FGD 2, Male, 48 years).

Concerning distance of CHPS Compounds from communities, some of the community members argued that the locations where the facilities were sited were far away from the communities. As such, community members usually found it difficult accessing them especially during emergency situations such as childbirth. The following quotes summarise these views:

The problem is that some of the facilities (CHPS compound) are sited a little bit too far away from the main community. Because of this when a woman is in labour somebody quickly goes to inform the TBA who is closer to them than the facility that somebody is in labour…It is only when they realise the situation is getting out of hand that the woman will be brought to the facility or you the health worker will be informed and go to help the woman

(FGD 1 Female, 33 years).

For me, where the facility has been sited is far from the village. People walk long distances and get there and they are told there is no medicine. So, before they will walk to Mafi Kumase, it disturbs them on the way. It happened once that someone was brought to the facility, but there was no medicine so they had to take the person back to Mafi Kumase, but before they could get there the person died.

(FGD 4, Female, 31 years).

On the challenge of inadequate funding/lack of resources, it was revealed that the central government makes no allocation to the DHDs for carrying out activities on a daily basis, unless for projects. This therefore, restricts their ability to carry out monitoring and evaluation of CHPS activities in the districts, especially when their internally generated funds are inadequate to do so. As a result of lack of adequate funding, most CHOs were placed at the CHPS compounds without the recommended 15 steps of CHPS training they were supposed to undergo. Some of the views of the participants during the FGDs are presented in the following quotes:

One major issue is that there is no funding that comes from government that is for training, okay. So, if you plan programmes to implement on CHPS, it is very difficult to execute them. At the beginning of every year, we always plan nice training schedules, meetings, write all sort of fancy things, but we don’t really do the training just because of funding. We then wait for programme-related training…CHPS+ is coming to do a training…like what you are doing now

(FGD 1, Male, 39 years).

We have placed them (CHOs) at the CHPs zone, but we’ve not given them that orientation as CHOs. Because, giving them that orientation as CHOs requires some resources as well. So, while those resources are not there we can’t say we cannot place them at the CHPs zone to be working. Even the few CHNs that we trained as CHOs now are CHNs that we placed at the CHPs zone

(FGD 3, Male, 36 years).

### Priority areas for capacity development

A key aspect of the research conducted was to identify priority areas for capacity development of the health workers. At the end of the discussions, a number of areas emerged. The major ones were; logistics management, community entry and engagement, emergency delivery, communicative skills, conflict resolution and management, data management, management and leadership skills, integrated management of neonatal and childhood illnesses, management of non-communicable and communicable diseases, managing referrals at the CHPS level, managing neglected tropical diseases in the communities, integrated diseases surveillance and response, logistics management, and resuscitation of newborns. The quotes that follow expatiate on the views expressed by the health workers regarding their priority training areas:

#### Logistics management

What I have to say is … about providing the tools or the resources to work with and then when we give them the tools we want them to manage it and account for them accordingly. They should be taken through how to take stock and keep proper document for those resources being provided. I mean management of equipment and stock. Yes! Yes! That is it

(FGD 1, Male, 41 years).

#### Community entry and engagement

One thing I will like to say is that we also need training in community entry and engagement. When we are actually given some skills, it will actually help us when it comes to our community mobilisation and engagement to actually help support the facilities when it comes to mobilising resources in the communities and then getting the needs for the facilities to actually enable us to work

(FGD 1, Male 38 years).

#### Emergency delivery

Although someone per the criteria of being a CHO cannot do normal delivery, I am of the view that if the CHOs could actually be trained with the skill to carry out emergency delivery so that anything that happens, they will be able to actually assist and not just always referring everything some of which the women even die before reaching the hospital

(FGD 1 Male, 39 years).

#### Data management

I need training in data utilisation or data management. Day in day out, we generate data, but we don’t have much knowledge on how to analyse the data and use it to make informed decision at our various facilities. So, if we have that training, it will help us to be able to use the data that we generate to make decision to improve upon our service delivery in the facility

(FGD 3, Male, 34 years).

#### Management and leadership

I identified leadership and management as a training need. In health care delivery, we happen to come in contact with humans at all levels and we play leadership roles at each of the various levels. So, I think it’s very important that we develop our leadership skills and then how we relate to each other because we are all one, and we come into contact with people from different backgrounds

(FGD 3, Male, 36 years).

#### Integrated management of neonatal and childhood illnesses (IMNCI)

Integrated Management of Neonatal and Childhood Illnesses is one of the basic things that we (health workers) have to do. So, cases that we have seen in our facilities, we need to quickly have identification of those cases and how we can manage them prudently before referrals to the next level. Once we are able to get all those skills, we can manage the illnesses when they happen. So, the training in IMNCI is very necessary

(FGD 1, Male, 33 years).

#### Resuscitation of newborns

Resuscitation (of newborns)! Very, very important! Because we all know the first minute of a baby is very, very important. But some of the babies come and you try everything and it fails. What should you do before getting to the next level? [ok] thank you

(FGD 3, Female, 31 years).

#### Management of non-communicable diseases

I want to learn more about the management of diabetes and then hypertension. In relation to diet and then the medical aspect as well. I want to know more about it because am a nutrition officer and because there are new things that are coming up which you need to update yourself on

(FGD 3, Male, 34 years).

### Stakeholder perspectives on improving the implementation of CHPS

The participants expressed their views regarding the mechanisms that could be adopted to address the various challenges that confronted the successful implementation of CHPS in the two SLDs. The major suggestions which came up were targeted at addressing the challenge of community lack of ownership of CHPS programmes, lack of community entry and engagement, and the non-availability of medicines. They also indicated that self-help is very essential in addressing the challenges that militate against the effective implementation of CHPS. The traditional leaders in the various communities were also called upon to rally the support of their people behind the health workers so as to ensure that CHPS is able to meet its core mandate of achieving health for all in Ghana.

#### Addressing the lack of ownership of CHPS programmes

Regarding the challenge of lack of ownership of CHPS programmes, it was suggested that the health workers should properly inform the communities and their leaders on programmes before rolling them out so as to gain their support for such programmes. On the part of the communities, the leaders were entreated to be holding durbars and sensitising their people on the need to get involved in CHPS activities to make them successful. The following quotes summarise their views:

Concerning the community members not being concerned about CHPS, I think all they need is to understand what is going on. They need to be engaged to understand what is going on. What is the whole programme (CHPS) about? So that when they understand, they will also own the programme to make it a better one

(FGD 1, Male, 41 years).

So, the only thing is that we have to be holding durbars and then sensitising our people that anytime there is a problem beyond the scope of the nurse, the best thing to do is to forget about the situation. We have to let them know the health system is for all of us and we have to help build it together

(FGD 4, Male, 47 years).

#### Addressing the lack of community entry and engagement

It was suggested that appropriate community entry procedures are to be followed if the activities of CHPS are expected to be successful. In the Ghanaian context, this means, seeing the chiefs and elders of the communities and briefing them first of all, on all pending programmes and seeking their support, and then engaging the rest of the community membership in order to ensure that all stakeholders are brought on board. The ensuing quotes summarise the suggestions given in this regard.

The community members, chiefs and people, they have their strengths and then they knowing what they can do to improve the health system. But, if they are not properly consulted and we are not able to mobilise them very well for them to understand their role when it comes to CHPs implementation, it means that our work may not go on well or we will not be able achieve our goals. So, we need to hammer (emphasise) more on that aspect

(FGD 1, Female, 31 years).

So, the major solution to this problem is that there should be a community entry and reengagement especially with the TBAs so that there will be this relationship between us and the TBAs. Anytime pregnant mothers go to TBAs then they will bring the pregnant mother to the facility and I think the delay in seeking appropriate care will be a thing of the past

(FGD 1, Female, 36 years).

#### Addressing the non-availability of medicines

The key suggestion given in this regard was for the government and the Ghana Health Service to ensure that the CHPS compounds are always supplied with the basic and essential medicines to encourage community members to continue utilising the CHPS compounds. The following quote summarises the views of the participants in this regard.

We plead that CHPS compounds are supplied with medicines always so that they can help us, before if conditions worsen, we can go forward (to higher levels of health care management like sub-district/district level health facilities)

(FGD 4, Female, 51 years).

#### Improving the delivery of CHPS through community self-help

It was revealed that some of the communities were already engaging in self-help projects with support from other stakeholders to make CHPS implementation successful. The other community stakeholders present were, therefore, entreated to replicate such gestures to ensure that the implementation of PHC through CHPS in their own communities is successful.

For us in Dalikpui CHPS zone, what we did is that they said that the contracts that came did not have enough money for us to do the work. So, we the community members ourselves fetched sand and started molding the blocks. Even as we speak now, we have bought some pebbles. So, if the work begins, we the community members are ready to help them in every aspect for the work to be done…We are supporting the assembly to build our CHPS block to completion

(FGD 4, Male, 46 years).

#### Addressing late reporting of cases

The issue of community engagement was again indicated as the panacea to the reduction of late reporting of cases which prevailed in the two SLDs. Once that is done, it was argued that the current number of reported mortalities especially those resulting from maternal complications, will significantly reduce.

If we are able to engage our community leaders and members effectively for them to take the facility deliveries seriously, I think the delays in seeking care will come down if not stopped at all. With that we would not be hearing anything about deaths here and there again because of childbirth

(FGD 1 Male, 36 years).

#### The role of traditional leaders (chiefs, queens, queen mothers, and elders)

Traditional leaders were also called upon to play their important roles as community focal persons to ensure that members participate fully in CHPS activities in order to make its implementation successful. When asked to indicate the role that he as well as other traditional leaders could play to improve the implementation of CHPS in his district, a chief participant had this to say:

Just as you have said, I have taken heed and I will work on it. I am also a chief. They also have chiefs. They are my heads. They are our leaders, aren’t they? Ahaa. If a good initiative has come, and I am taking care of it and I am benefiting from it and I bring this initiative to you and you don’t take good care of it then… I will take it to them and I will discuss it with them if they take it then fair enough.

(GD1, Male, 56 years).

## Discussion

The conceptual framework of the study posits that for the implementation of any PHC programme to be successful, it must be guided by political commitment, integration of promotive, curative, preventive health care services, equity, accessibility, affordability, availability, effectiveness, and efficiency [4,19]. We, however, found that most of these principles in the conceptual framework were missing in the SLDs. For instance, that no funds were usually earmarked for the District Health Directorates (DHDs) (bodies mandated to oversee all health-related activities in Ghanaian administrative districts) to carry out CHPS activities by successive governments, is indicative of a lack of political will to invest in the health of the people. The responsibility is thus, left entirely on the Ghana Health Service to look for funders to support primary health care in the country; like the CHPS+ project which led to the conduct of the current research. The implication of this is that once funding is unavailable, PHC through CHPS comes to a standstill.

It was interesting to realise that in the present study, CHOs who were placed in the CHPS compounds were not trained on CHPS implementation before being placed due to lack of funding. This points to a major deficit in the training of health professionals on-the-job in order to make them system-ready for delivering services to the community members. This finding comes at the backdrop of previous studies which have established the important role of on-the-job training [25,26].

The conceptual framework of the study argues that for PHC to be successful, patients should have timely and affordable access to PHC that is geographically convenient [21]. We, however, found in our study that not only were CHOs not available to provide care when needed, location of CHPS compounds were equally geographically inconvenient for the community members who are the expected beneficiaries, especially during emergencies. An implication of this finding was that the community members, especially pregnant women mainly resorted to the utilisation of Traditional Birth Attendants (TBAs) during delivery. This comes at the backdrop that home deliveries with TBAs usually come with high maternal and neonatal mortality especially when they are untrained [27–30] which the participants in our study strongly alluded to. What makes the situation worse is that due to improper community entry, TBAs in our study were usually unwilling to refer cases they were unable to handle for skilled orthodox care and thereby increasing the chances of maternal deaths. The important role of community entry and engagement as realised in previous studies [31–35] is, therefore, confirmed by our findings.

The conceptual framework recognises drugs supply and availability as pre-requisites for the effective implementation of PHC [21]. In our study, we found that basic medicines for treating patients were usually non-existent. Community members were constantly requested to go and buy their own medications after being attended to by the CHOs/CHNs. This does not auger well for the health system as it would eventually lead to declined skilled health care utilisation as found in previous studies [36,37] and eventually inhibit the country’s targets of achieving health for all using CHPS as a PHC intervention.

For CHPS to be effective, the conceptual framework recognises the important role of patient-provider respect and trust as well as community engagement [21]. These principles were, however, generally missing. Our findings for instance, revealed that some CHOs/CHNs exhibited negative attitudes towards community members/patients. They also did not appropriately recognise the authorities of the communities (especially chiefs and elders) by informing and adequately engaging them prior to the execution of the CHPS programmes and activities. Thus, community entry was generally lacking. The result was a general apathy on the part of the community members towards CHPS programmes and activities, which is detrimental to its efficient implementation.

## Strengths and limitations

A major strength of this study was deployment of the scorecards. The scorecards clearly elucidated key issues for discussion in the FGDs. They made it simple for the FGD participants to organise their thoughts for the discussions. This enabled the major issues of importance to come out clearly during the discussions. The study also conducted separate FGDs for community members and health workers before finally merging the two groups for the general discussions. This approach ensured that the various groups, without any influence or intimidation, were able to bring out their concerns for discussion.

Despite the strengths and important findings made in the current study, it is worth mentioning its potential limitations. As a qualitative study, our research relied on the views of participants for discussions and conclusions. While we recognise that appropriate stakeholders were involved in the FGDs and GDs, their personal biases could have influenced the views expressed. The participatory nature of the study, where all stakeholders were brought together after their respective FGDs, however, ensured that consensus was reached on the major challenges in the implementation of CHPS, priority areas for capacity development of frontline health workers, and appropriate strategies for addressing challenges identified. Another gap in our study was our inability to extensively explore the availability and access to effective CHPS services nothing. This could, therefore, be the focus of future studies.

## Conclusion

Health-worker, community, and health systems-based challenges inhibit the implementation of CHPS in Ghana. Capacity development of health professionals and continuous community engagement are, however, avenues that can improve implementation of the programme.

## Recommendations

To ensure that CHPS is implemented effectively to achieve its overarching goal of achieving health for all, the study highlighted that health workers need to always properly inform the communities they serve and their leaders on CHPS activities before rolling them out so as to gain their support. The study also highlighted the need for the Ghana Health Service and the District/Municipal/Metropolitan health directorates to ensure that the CHPS compounds are always supplied with the basic and essential medicines.

The findings made regarding the priority areas of capacity development for frontline health workers in the face of challenges found during the stakeholder discussions justify the essential need for the CHPS+ project as well as government and other stakeholders to immediately organise capacity development trainings for the workers with support from the Ghana Health Service and the other partners. Areas of focus for such trainings could be logistics management, community entry and engagement, emergency delivery, communicative skills, conflict resolution and management, data management, management and leadership skills, integrated management of neonatal and childhood illnesses, management of non-communicable and communicable diseases, managing referrals at the CHPS compounds, managing neglected tropical diseases in the communities, integrated diseases surveillance and response, logistics management, and resuscitation of newborns.

## Abbreviations

CDC: Centres for Disease Control and Prevention
CHPS: Community Based Health Planning and Services
DANIDA: Danish International Development Agency
DHDs: District Health Directorates
DM: Data Manager
EMIS: Education Management Information System
FTI: Faecally Transmitted Infections
HWWS: Hand Washing With Soap
JHS: Junior High School
NTDs: Neglected Tropical Diseases
PPP: Public Private Partnership
RHDs: Regional Health Directorates
SDG: Sustainable Development Goals
SHEP: School Health Education Programme
SLD: Systems Learning District
SPH: School of Public Health
UHAS: University of Health and Allied Sciences
UNICEF: United Nations Children Fund
WASH: Water, Sanitation, and Hygiene
WHO: World Health Organization
